# Complete Genome Sequence of the Oncolytic *Sendai virus* Strain Moscow

**DOI:** 10.1128/genomeA.00818-16

**Published:** 2016-08-11

**Authors:** Sergei S. Zainutdinov, Artem Y. Tikunov, Olga V. Matveeva, Sergei V. Netesov, Galina V. Kochneva

**Affiliations:** a Novosibirsk State University, Novosibirsk, Russia; b State Research Center of Virology and Biotechnology Vector, Koltsovo, Novosibirsk Region, Russia; c Institute of Chemical Biology and Fundamental Medicine of SB RAS, Novosibirsk, Russia; d Biopolymer Design LLC, Acton, Massachusetts, USA

## Abstract

We report here the complete genome sequence of *Sendai virus* Moscow strain. Anecdotal evidence for the efficacy of oncolytic virotherapy exists for this strain. The RNA genome of the Moscow strain is 15,384 nucleotides in length and differs from the nearest strain, BB1, by 18 nucleotides and 11 amino acids.

## GENOME ANNOUNCEMENT

Cancer cells commonly acquire sensitivity to viruses due to the genetic loss of antiviral resistance mechanisms and impaired regulation of biosynthesis (1). The use of oncolytic viruses in cancer therapy raises the issue of their safety to people and the environment. The *Sendai murine paramyxovirus* is nonpathogenic for humans and farm animals but causes respiratory illness in mice. *Sendai virus* kills malignant cells in preference to normal cells (2, 3). The death of cancer cells results from both direct action of the virus and virus-induced activation of anticancer immunity. *Sendai virus* induces the formation of syncytia, which promote viral spread within a tumor by protecting the virus from exposure to host-neutralizing antibodies. As a result, *Sendai virus* can cause mass killing of malignant cells and thus tumor destruction. This virus is also a powerful inducer of interferon and other cytokines that promote antitumor immunity (3).

In the late 1950s, V. M. Zhdanov from the Ivanovsky Institute of Virology (Russia) obtained *Sendai virus* from Japan (4). Then, the virus was transferred to V. M. Senin at the Blokhin Russian Cancer Research Center (Moscow) and underwent about 30 passages in chick embryos. The resultant strain was given the strain name Moscow. In the 1990s, this strain was tested as an experimental oncolytic agent on volunteer patients in Moscow and St. Petersburg hospitals who had various stage III and IV malignancies. Although in some patients improvement was transient or not observed at all, other patients achieved long-term remission. In these cases, resorption of primary tumors and metastases was observed after one or two courses of Moscow strain therapy, and no signs of the disease were discovered even after five to 10 years or more (2, 5, 6).

The *Sendai virus* Moscow strain used in these clinical tests was deposited in the American Type Culture Collection as PTA-13024 and PTA-121432. PTA-13024 contains the virus in frozen allantoic fluid, and PTA-121432 contains the virus in lyophilized form. To determine its complete genome sequence, 32 pairs of oligonucleotide primers were used to amplify different regions of viral RNA by reverse transcription-PCR (RT-PCR). The PCR products were purified and sequenced with an automated sequencer (Genetic Analyzer 3500; Applied Biosystems).

The software package MEGA version 6 was used to assemble the viral genome of 15,384 nucleotides (nt). The genome was built by overlapping (overlap, ~300 nt) sequenced PCR fragments of ~800 nt. A GenBank homology search revealed that a top hit for the newly sequenced strain Moscow is strain BB1 (accession no. DQ219803); only 18 mismatches were found between the two strains, and seven out of the 18 mismatches were synonymous for amino acids. Other *Sendai virus* genomes deposited in GenBank (accession numbers AB005796, AB039658, AB275416, AB065187, AB065186, AB065189, AB065188, M30203, AB195968, AB275417, NC_001552, AB195967, M69046, EF679198, M30204, and M30202) were less similar to the Moscow strain. At least 700 mismatches were detected between the Moscow strain genomic sequence and any of the genomic sequences of the strains from the list above.

### Accession number(s).

This genome sequence has been deposited in GenBank under the accession no. KP717417.
